# Twelfth International Medical Congress, Moscow, Russia, August 19-26, 1897

**Published:** 1897-05

**Authors:** 


### TWELFTH INTERNATIONAL MEDICAL CONGRESS, MOSCOW, RUSSIA, AUGUST 19—26, 1897.

In a letter dated Moscow, February 14th, the Secretary-general, Prof. W. K. Roth, communicates the following facts for the information of the American physicians who intend to participate in the twelfth International Congress which is to be held at Moscow from August the 19th to 26th.

The Trans-Atlantic Steamship Companies refuse one and all any reduction of the usual charges. In their replies, most of which are couched in courteous language (the originals are in the possession of the undersigned), they admit the existence of a trust, or contract, or agreement which prevents them from lowering their prices; a few are so polite as to express their regrets.

Reductions of fares on Russian railroads are expected shortly. The French, Spanish, Swedish, and Hungarian railroads promise a reduction of 50 per cent; so do the Italian, for a distance of 500 kilometres; less (down to 30 per cent.) for shorter distances. The Mediterranean lines (Messageries Maritimes, General Italian Navigation Company, Austrian Lloyd) grant from 25 to 50 per cent.

The undersigned chairman is not authorized to issue certificates of any kind in favor of congressists. He will try to ascertain, however, in which way their movements may be facilitated, and may receive a reply in the second half of April. Extracts of papers to be read before any of the sections ought to reach the Secretary-general before June 1st in order to be printed in the preliminary volume.

A special prospectus containing the final details referring to traveling, lodging, festivities, etc., is promised for a near future. It will be communicated at once to the medical journals, and to the press of the country.

A. JACOBI,
Chairman American National Committee.

AMERICAN NATIONAL COMMITTEE

J. S. BILLINGS, M. D., New York.

FRANK P. FOSTER, M. D., New York.

CLAUDIUS H. MASTIN, M. D., Mobile.

S. WEIR MITCHELL, M. D., Phila.

CHARLES A. L. REED, Cincinnati.

GEORGE B. SHATTUCK, M. D., Boston.

F. J. SHEPHERD, M. D., Montreal.

GEO. F. SHRADY, M. D., New York.

W. S. THAYER, M. D., Baltimore.

A. JACOBI, M. D., 110 West 34th St., New York, *Chairman.*

